# Beyond digital twins: the role of foundation models in enhancing the interpretability of multiomics modalities in precision medicine

**DOI:** 10.1002/2211-5463.70003

**Published:** 2025-02-24

**Authors:** Sakhaa Alsaedi, Xin Gao, Takashi Gojobori

**Affiliations:** ^1^ Computer Science, Division of Computer, Electrical and Mathematical Sciences and Engineering (CEMSE) King Abdullah University of Science and Technology (KAUST) Thuwal Saudi Arabia; ^2^ Center of Excellence on Smart Health King Abdullah University of Science and Technology (KAUST) Thuwal Saudi Arabia; ^3^ Center of Excellence for Generative AI King Abdullah University of Science and Technology (KAUST) Thuwal Saudi Arabia; ^4^ College of Computer Science and Engineering (CCSE) Taibah University Madinah Saudi Arabia; ^5^ Biological and Environmental Sciences and Engineering King Abdullah University of Science and Technology (KAUST) Thuwal Saudi Arabia; ^6^ Marine Open Innovation Institute (MaOI) Shizuoka Japan

**Keywords:** bioinformatics, biomedicine, digital twins, foundation models, LLM, multiomics

## Abstract

Medical digital twins (MDTs) are virtual representations of patients that simulate the biological, physiological, and clinical processes of individuals to enable personalized medicine. With the increasing complexity of omics data, particularly multiomics, there is a growing need for advanced computational frameworks to interpret these data effectively. Foundation models (FMs), large‐scale machine learning models pretrained on diverse data types, have recently emerged as powerful tools for improving data interpretability and decision‐making in precision medicine. This review discusses the integration of FMs into MDT systems, particularly their role in enhancing the interpretability of multiomics data. We examine current challenges, recent advancements, and future opportunities in leveraging FMs for multiomics analysis in MDTs, with a focus on their application in precision medicine.

## Graphical abstract

This review highlights how foundation models enhance predictive healthcare by integrating advanced digital twin modeling with multiomics and biomedical data. This approach supports disease management, risk assessment, and personalized medicine, with the goal of optimizing health outcomes through adaptive, interpretable digital simulations, accessible across diverse healthcare settings.

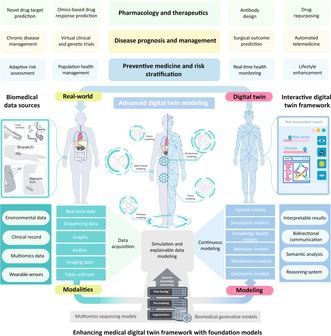

**Abbreviations**
AI: artificial intelligence
BERT: bidirectional encoder representations from transformers
CNNs: convolutional neural networks
COPD: chronic obstructive pulmonary disease
DL: deep learning
DT: digital twins
EHR: electronic health records
FM: foundation models
GPT: generative pretrained transformer
IoT: internet of things
LLM: large language model
MCGAN: medical conditional generative adversarial network
MDT: medical digital twin
ML: machine learning
NER: named entity recognition
PLM: product lifecycle management
QR: question answering
RE: relation extraction
UMLS: unified medical language system
VR: virtual reality

The concept of a medical digital twin (MDT) has gained traction as a key component of precision medicine [1]. The MDT is a dynamic, continuously updated model that integrates data from various sources, including clinical, molecular, and imaging data, to simulate the health trajectory of a patient [2, 3]. One of the most promising applications of MDT in precision medicine lies in the integration of multiomics data—genomics, transcriptomics, proteomics, metabolomics, and epigenomics, to provide a holistic view of a patient's biology [3]. Several applications in MDTs use multiomics data to enhance medical decision‐making, provide personalized treatment plans, and simulate drug experiments. These approaches help optimize treatment plans, select therapies and drugs, and improve surgical planning with augmented reality tools [3, 4, 5]. However, interpreting complex datasets from biomedical modalities, such as multiomics, medical imaging, and electronic records, presents significant challenges, particularly in terms of interoperability and producing accurate simulations [1, 6]. Most existing MDT models rely on one‐way data transfer from physical entities to virtual models and often overlook environmental factors, resulting in inaccurate virtual representations [6, 7]. With the growing influence of artificial intelligence (AI), models have increasingly enhanced data representation and improved the performance of downstream tasks across various fields, including medicine [8]. Foundation models (FM), which are advanced machine learning models pretrained on massive datasets and adaptable to various tasks, offer a promising solution to these limitations [9]. This review is one of the first to focus on how FMs and their applications can enhance the interpretability of multiomics data in MDTs and their potential to advance precision medicine. Furthermore, it addresses the future challenges and limitations of FMs, highlighting directions for future research to optimize their use in MDTs for improving precision medicine.

## Search strategy

In July 2024, we reviewed 930 English‐language articles from databases including Google Scholar, PubMed, Web of Science, and Scopus. We used search terms such as ‘Digital twin’, ‘Digital twin and multiomics’, and ‘Digital twin and omics’. The articles selected for this review included original research, reviews, viewpoints, and opinion pieces. We excluded 470 articles that were brief or not specifically related to digital twins (DTs). For articles on FMs, we used a similar approach but also included papers from arXiv, as this is a newer field with fewer publications under the keywords ‘Foundation models in precision medicine’, ‘Generative models in multiomics and digital twin’, and ‘LLMs in medicine and digital twin’. The literature on generative and FMs was reviewed from 2020 to 2024, covering relevant studies since their inception.

## History of digital twins

The concept of DTs has a rich history that spans multiple industries and decades [1, 10]. It first emerged in the 1960s when NASA pioneered the use of digital models to simulate the conditions on the moon's surface for the Apollo missions [11]. This early adoption showcased the power of virtual replicas for enhancing mission safety and success [11]. By the 1970s, the oil and gas industry began leveraging DTs to explore new energy reserves, and theautomotive industry embraced the technology to optimize vehicle design and testing [12].

In the 1990s, DTs gained more widespread recognition with their mention in David Gelernter's book, ‘Mirror Worlds’, which explored the idea of creating virtual representations of real‐world systems [13]. In 2002, Dr. Michael Grieves introduced the concept of the DTs in the context of product lifecycle management (PLM) at NASA, laying the foundation for future applications [1, 10]. As the 2000s unfolded, the concept expanded into manufacturing and other industries, with real‐time data and statistical models being employed to create digital counterparts for physical systems [10, 14]. These twins were used to optimize processes, monitor systems, and predict failures in real‐time. The 2010s saw the introduction of internet of things (IoT) devices, wearables, and deep learning into healthcare, allowing DTs to be applied to complex medical problems [10]. This decade marked a significant shift in the adoption of DTs for patient simulations, disease prediction, and personalized medicine.

### Medical digital twin

By the 2000s, DTs began emerging in medicine, focusing on medical device simulations. In healthcare, DTs started incorporating statistical models to predict patient outcomes using structured data such as electronic health records (EHRs) and clinical data. These early models were primarily used for retrospective analysis with limited predictive capabilities [15].

By the 2010s, the rise of machine learning (ML) advanced DTs further by enabling more sophisticated data analysis and pattern recognition, especially for complex datasets such as genomics and imaging [4]. ML models significantly improved predictive accuracy in disease diagnosis and treatment outcomes, though they still relied heavily on structured data.

Between 2015 and 2018, the emergence of deep learning (DL) transformed DTs into more dynamic systems, capable of handling large‐scale unstructured data such as medical images and clinical text [4]. Deep learning models facilitated automated feature extraction from complex multimodal datasets, enhancing predictions in areas such as cancer and cardiovascular diseases [16].

By early 2022, the integration of generative models introduced new capabilities for simulating complex medical scenarios, including drug interactions and personalized healthcare strategies. Large language models (LLMs) began improving DTs by enabling more effective handling of unstructured text data, such as clinical notes and medical literature [4, 17]. By the end of 2023, FMs further advanced DTs by enabling highly personalized treatment planning and precision medicine [16].

**Fig. 1:**
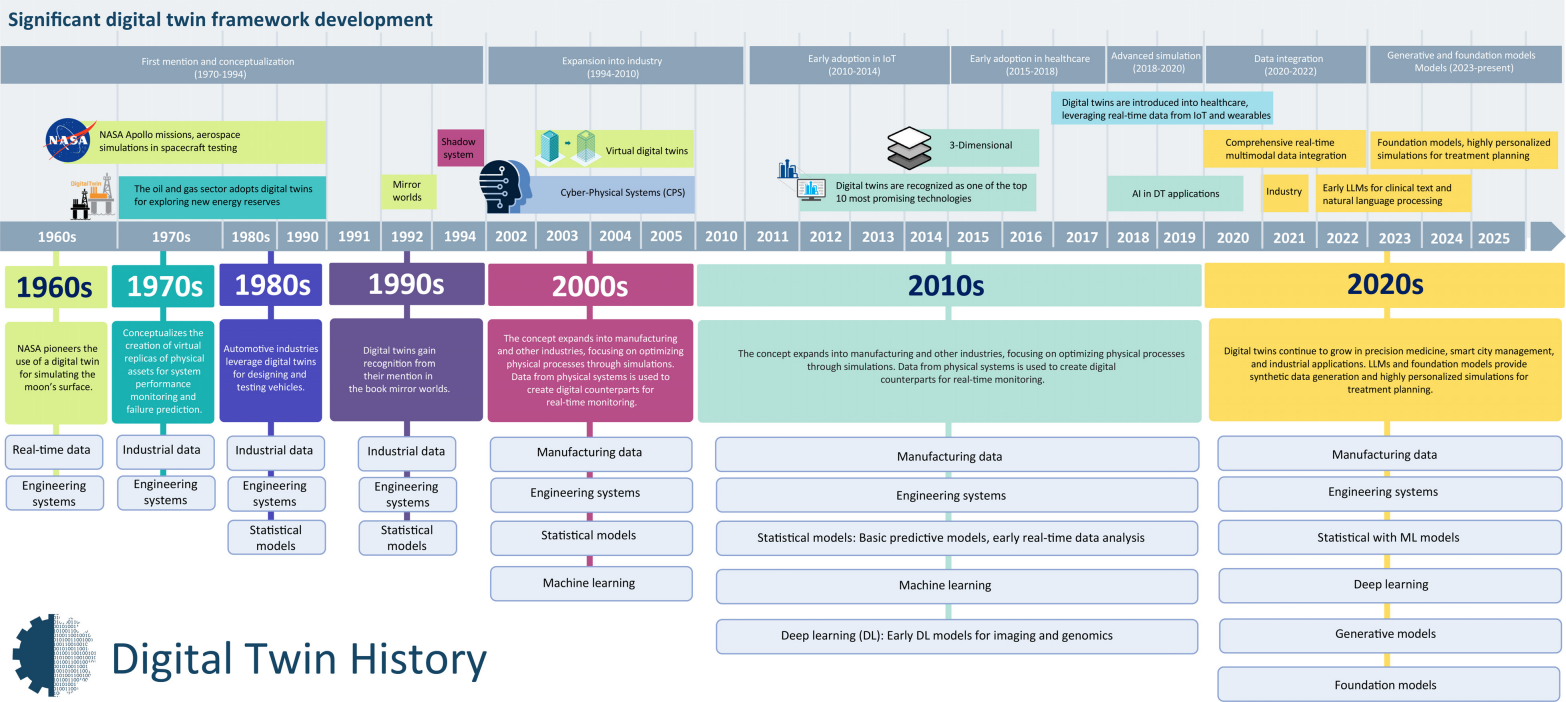
Development of digital twin technology across industries. This figure illustrates the historical progression of digital twin technology from its origins in the 1960s with NASA's moon simulations to its widespread application in industries such as oil and gas, automotive, and manufacturing by the 1970s–2000s. In the 2010s, the integration of IoT devices and wearables brought digital twin into healthcare for personalized medicine and real‐time patient monitoring. By the 2020s, AI‐driven foundation models and large language models enhanced the capabilities of digital twin, enabling more comprehensive simulations for healthcare, smart cities, and industrial process optimization. The timeline reflects key innovations that contributed to the evolution of digital twin across these sectors.

The development of FMs is expected to continue, further enhancing digital twin applications in real‐time data processing, disease progression simulations, and even more precise, individualized healthcare solutions [4]. Figure 1 illustrates the evolution of DTs, highlighting significant milestones in their development throughout this century.

## Key criteria for medical digital twins

Although many studies have explored MDTs in personalized medicine, clear criteria are essential for determining whether an implemented system qualifies as a true digital twin [18]. Based on recent research and expert consensus, the following key criteria must be met for a system to be considered DTs in the medical domain:

- Real‐time data integration: The system should continuously integrate real‐time data from diverse sources such as EHRs, wearable devices, or omics technologies [18]. This allows dynamic updates and adjustments to reflect the patient's current condition accurately [19].
- Simulation and predictive modeling: A core component of MDT is the ability to simulate disease progression, treatment outcomes, or patient‐specific health scenarios [18]. Advanced AI algorithms employed to predict future states of patient health, supporting proactive care.
- Individualized and adaptive models: The system should create a highly personalized digital representation of the patient, which adapts over time as new data becomes available [18]. This ensures that predictions and simulations remain relevant and continuously improve in accuracy, reflecting the evolving nature of the patient's health [20].
- Bidirectional communication: The MDT must enable bidirectional feedback [18]. Not only should it simulate patient conditions and forecast outcomes, but it must also provide real‐time, actionable insights that clinicians can use to inform actual treatment plans.
- Decision support system: The model must assist clinicians by offering personalized treatment recommendations or risk assessments, based on data‐driven analyses [18, 20]. This capability enhances the overall quality of care by supporting more informed and precise medical decisions.

These criteria not only define the core functionality of MDTs but are also flexible enough to allow for scaling and enhancement. As new AI models, such as FMs [21], are integrated, they continue to improve the accuracy and performance of MDTs.

## Foundation models

Foundation models are large‐scale AI models pretrained on massive datasets, making them highly adaptable to a variety of downstream tasks, including biomedical tasks [21]. Built on deep learning architectures such as transformers, these models serve as the backbone for more specialized AI applications [22]. By learning general patterns during pretraining, FMs can be fine‐tuned for specific tasks, including text generation, image analysis, and multimodal tasks that integrate various data modalities FMs, particularly LLMs, applied to multiomics such as text, images, and clinical data [21]. Notable examples of transformers include generative pretrained transformer (GPT) [23] and bidirectional encoder representations from transformers (BERT) [24], which are trained on diverse datasets and adapted for specific applications.

These transformers considered as LLMs a subset of FMs, specialize in tasks related to text understanding, generation, summarization, and question answering [25]. LLMs are invaluable in medical contexts, particularly for interpreting multiomics data in MDTs [25]. They enhance the ability of MDTs to interpret complex medical data, draw causal inferences, and incorporate knowledge from scientific literature, overcoming previous limitations in data interpretation [26].

Key strengths of FMs include transfer learning, where pretrained models are fine‐tuned on domain‐specific datasets, such as biomedical data, allowing efficient adaptation [21, 27]. Additionally, multi‐task learning enables these models to handle diverse tasks, including classification, regression, and clustering, making them highly versatile in managing complex omics data. Scalability is another advantage, as FMs are capable of processing vast datasets, making them ideal for multiomics integration [21].

## Enhancing medical digital twins with foundation models

Recent methods in FMs play a crucial role in addressing the challenges faced by MDTs, such as analyzing and integrating medical and multiomics modalities, providing explainable and accurate digital simulations, ensuring interpretable system outcomes, and facilitating understandable bidirectional communication.

### Multiomics sequencing models

Sequencing data excel in analyzing and integrating diverse data types by harmonizing and processing them [28, 29, 30]. Several LLM models have been pretrained on datasets spanning multiple species to solve various biocomputational tasks for enhancing omics sequence analysis and interpretation in bioinformatics and medical fields [28, 29, 31]. The following LLM models demonstrate how LLMs contribute to omics analysis and enhance data integration in MDTs:

- Genomics: In DNA sequence generation, significant progress has been made with the introduction of pretrained models such as DNAGPT [32]. Complementing this, the nucleotide transformer serves as another foundational model for advancing genomic phenotype prediction [33]. In predicting cis‐regulatory sequences, DNABERT‐2 [34, 35], iEnhancer‐BERT [36], and BERT‐Promoter [37] have significantly impacted the prediction of regulatory element sequences and positions, crucial for development and physiology [29]. GROVER aids in identifying structures within genomic regions essential for functional genomics annotations [38]. Another model, TFBert, effectively identifies DNA–protein interactions using 690 ChIP‐seq datasets [39].
- Transcriptomics: The integration of LLMs into transcriptomics offers valuable insights into RNA sequence analysis. For example, RNA‐FM uses self‐supervised learning to predict RNA secondary and tertiary structures from 23 million noncoding RNA sequences [40], while RNA‐MSM predicts 2D base pairing probabilities and 1D solvent accessibilities using homologous sequences from RNAcmap [41]. RNABERT specializes in RNA secondary structure prediction and family classification, aiding in transcript annotation [42]. SpliceBERT enhances RNA splicing prediction by modeling precursor mRNA sequences, improving our understanding of splicing disruptions [43]. In single‐cell transcriptomics, tGPT captures features for cell clustering in large datasets such as the Human Cell Atlas, while scFoundation, with over 100 million parameters, improves gene expression prediction and clustering using read‐depth‐aware modeling [44]. Moreover, FMs such as GenePT identify cell types and trace lineage relationships, advancing the analysis of cellular diversity and developmental pathways [45].
  For gene expression prediction, LLMs analyze RNA modifications. The BERT‐m7G model identifies N7‐methylguanosine sites [46], while Bert2Ome combines BERT with convolutional neural networks (CNNs) to detect 2′‐O‐methylation sites [47]. These models offer cost‐effective alternatives to traditional methods, enhancing our understanding of RNA modifications and their role in gene expression.
- Proteomics: The integration of LLMs into proteomics represents a significant advancement in bioinformatics by enabling the creation of DTs of biological systems. These digital representations facilitate advanced analysis and interpretation of protein data at an unprecedented scale, aiding in the prediction of protein structures and the annotation of protein functions. For example, TAPE [48] set a benchmark for protein transfer learning by defining tasks, datasets, and metrics for structural prediction and design, enhancing protein sequence analysis through self‐supervised pretraining. LLMs such as ProtTrans [49], UDSMProt [50], and UniRep [51] predict 3D protein structures from amino acid sequences, which is key for understanding protein function, drug discovery, and design. However, these models often overlook protein‐specific features [52], indicating the need for further refinement to fully realize their potential in developing accurate DTs of biological entities.
  In predicting and analyzing protein function, PromptProtein [53] is a leading prompt‐based model that excels in multi‐task pretraining and fine‐tuning for protein analysis, outperforming existing methods in predicting protein functions and biophysical properties. Similarly, ProteinBert [54], pretrained on 106 million proteins, focuses on protein function prediction, effectively capturing both local and global protein features.
- Epigenomics: Epigenetics involves changes in gene activity that do not involve alterations to the genetic code itself but can be inherited. LLMs can predict the locations and effects of epigenetic modifications, such as DNA methylation, which influence gene expression and contribute to understanding complex traits and diseases. In oncology, DNA methylation is crucial for gene regulation and serves as a biomarker for metagenomic binning. LLMs such as BERT6mA [55], iDNA‐ABT [56], and MuLan‐Methyl [57] have been developed for predicting various methylation types. iDNA‐ABT improves species identification and MuLan‐Methyl predicts methylation sites using multiple transformer models. LLMs are also applied to predict long noncoding RNAs involved in cancer. LncCat using category boosting and ORF‐attention features, enhances the identification of regulatory lncRNAs and small open reading frames, which encode functional peptides [58].

### Biomedical generative and language models

FMs are transforming biomedical data analysis by facilitating the extraction of insights from both structured datasets and unstructured clinical narratives. By integrating real‐time data from sources such as EHRs, clinical notes, and omics profiles, these models continuously update the patient's digital twin, dynamically reflecting their health status. The ability to identify relevant biomarkers and critical patient‐specific patterns significantly enhances personalized medicine.

- Biomedical language models: Pretrained models such as BioBERT [59] and PubMedBERT [60] have been designed specifically for biomedical literature and clinical text analysis. These models excel at tasks such as named entity recognition (NER), relation extraction (RE), and question answering (QA). For instance, BioBERT has been successfully applied to the extraction of disease mentions, drug interactions, and gene‐disease associations from EHRs and scientific publications. Additionally, PubMedBERT is instrumental in summarizing biomedical research findings for clinicians and identifying adverse drug reactions, contributing to better clinical decision‐making in real time [61]. These language models enable clinicians to stay updated with the latest research and apply evidence‐based insights directly to patient care.
- Medical language models: ClinicalBERT [62] and UmlsBERT [63] are designed to handle clinical data from sources such as the MIMIC‐III database and other EHR systems. These models enhance the analysis of patient‐specific health records by performing clinical concept extraction and disease prediction. For example, ClinicalBERT has been used to predict patient readmission rates and mortality based on EHR data, allowing hospitals to identify high‐risk patients and allocate resources more effectively. UmlsBERT, by incorporating the unified medical language system (UMLS), improves the extraction of medical terminologies, assisting in diagnosing conditions based on symptoms and personalizing treatment strategies. These models are crucial for parsing complex medical histories and interpreting medication explanations for enhanced patient care [64].
- Biomedical generative models: Generative models such as Dr. Variational autoencoder (Dr.VAE) [65] and medical conditional generative adversarial network (MCGAN) [66] are key players in personalized medicine, particularly in predicting patient‐specific responses to treatments and generating therapeutic interventions tailored to individual genetic and clinical profiles. For instance, Dr.VAE has been applied to simulate drug response scenarios in cancer patients, facilitating the customization of chemotherapy regimens. Additionally, MCGAN generates synthetic patient data that mirrors real‐world cohorts, which is invaluable in rare disease studies where patient samples are limited. These models overcome data scarcity and provide new pathways for treatment design and validation, enabling more accurate simulations of drug interactions and patient outcomes in conditions such as diabetes and oncology [67].

### Simulation and explainable decision support systems

FMs enhance the decision‐making process in healthcare by generating interpretable outputs that help clinicians navigate complex biological data. These models are particularly useful in supporting explainable AI, which ensures that clinicians can understand and trust the decisions made by AI systems [68, 69]. FMs not only simulate patient outcomes but also provide interactive interfaces that allow healthcare professionals to visualize and adjust parameters in real time [70].

- Simulation and predictive modeling: Models such as LIME [70] and SHAP [71] are essential tools in explainable AI, providing explanations for the predictions made by machine learning models. These techniques are used extensively in healthcare to interpret predictions from complex models. For example, SHAP has been employed in cardiovascular risk assessments to explain how individual patient features, such as cholesterol levels and smoking history—contribute to the overall risk of heart disease [68]. Similarly, LIME allows clinicians to visualize the impact of specific variables on disease outcomes, offering transparency in decision support tools.
- Interactive and explainable decision support systems: FMs with statistical models enhance bidirectional communication between AI systems and healthcare providers. For instance, What‐If Tool [72] enables clinicians to interactively explore model predictions under different scenarios, such as adjusting a patient's medication dosage or lifestyle factors, to observe potential outcomes. This level of interaction fosters a more informed decision‐making process, where clinicians can engage with AI systems to refine and personalize treatment plans. Additionally, models such as InterpretML [73, 74] provide a range of algorithms for model interpretation, allowing clinicians to visualize decision‐making processes and gain insights into model behavior. Interactive dashboards powered by FMs help summarize risk assessments and suggest potential treatment options in a manner that is intuitive for clinical use [69, 75].
- Dynamic clinical insights: By generating human‐readable explanation, FMs provide actionable insights that translate complex biological data into easily understandable formats [69]. For example, in semantic reasoning applications, FMs generate real‐time feedback on patient diagnoses and treatment effectiveness. They provide summaries of potential interventions in user‐friendly language, which helps in identifying optimal treatment pathways. The ability to offer real‐time, explainable insights allows clinicians to adjust their strategies based on the evolving condition of the patient's digital twin, leading to more accurate and personalized healthcare recommendations [76].

Table 1 provides a comprehensive overview of FMs in bioinformatics and clinical applications. The integration of FM into biomedical workflows has led to notable improvements in disease prediction, treatment personalization, and data‐driven decision‐making that support MDT applications.

**Table 1:** Comprehensive overview of foundation models in biomedicine and medical digital twins.

Model name	Data sources	Model architecture	Biological problem/task	References
Bioinformatics and biological sequencing models				
DNABERT	Human genome	Transformer	DNA sequence analysis	[34]
DNABERT‐2	Human genome	BERT	Cis‐regulatory sequence prediction	[35]
DNAGPT	Human genome	GPT‐such as	DNA sequence generation	[32]
Nucleotide transformer	Human genome	Transformer	Genomic phenotype prediction	[77]
iEnhancer‐BERT	Human genome	BERT	Cis‐regulatory sequence prediction	[78]
BERT‐Promoter	Human genome	BERT	Promoter sequence and position prediction	[37]
GROVER	Human genome	Transformer	Genome annotation	[38]
GPN	Genomic datasets	Transformer	Causal variant prediction and rare variant analysis	[79]
BERT6mA	Genomic datasets	BERT	DNA methylation (6 mA) site prediction	[55]
iDNA‐ABT	Genomic datasets	Transformer	DNA methylation prediction with species identification	[56]
Transcriptomics models				
RNA‐FM	Noncoding RNA dataset	Transformer	RNA secondary and tertiary structure prediction	[41]
RNA‐MSM	RNAcmap homologous sequences	Transformer	RNA base pairing and solvent accessibility prediction	[32]
RNABERT	Noncoding RNA	BERT	RNA secondary structure prediction and family classification	[42]
SpliceBERT	Precursor mRNA sequences	BERT	RNA splicing prediction	[43]
LncCat	Noncoding RNA sequences	BERT	Long noncoding RNA and small ORF prediction	[58]
BERT‐m7G	RNA sequences	BERT	N7‐methylguanosine (m7G) site prediction	[32]
Bert2Ome	RNA sequences	BERT + CNN	2′‐O‐methylation site prediction	[47]
Proteomics models				
ProSTAGE	Protein datasets	Transformer	Prediction of mutation effects on protein stability	[80]
ProteinBERT	UniRef, Gene Ontology	BERT	Protein function prediction and GO annotation	[81]
ProtTrans	UniRef, UniParc, Big Fantastic Database	BERT, T5, Transformer‐XL, Albert, Electra, XLNet	Protein structure prediction	[82]
AlphaFold	Protein databases (PDB, UniProt)	Deep Learning	Protein structure prediction	[83]
ESM‐1bTransformer	UniParc, EvMutation	Transformer	Protein structure analysis	[84]
MSA Transformer	UniRef	Transformer	Protein sequence analysis	[85]
ProtGPT2	UniProt, ProteinGym	GPT‐2	Protein sequence design	[86]
SignalP 6.0	UniProt, PROSITE, TOPDB	ProtBERT + CRF	Signal peptide prediction	[87]
Tranception	UniProt, ProteinGym	Autoregressive Transformer	Fitness landscape prediction	[88]
TMbed	OPM, SignalP 6.0	ProtST + CNN	Transmembrane prediction	[89]
ProLLM	BIOSNAP	ProtT5	Inputting protein embeddings	[90]
TFBert	ChIP‐seq data	Transformer	DNA–protein interaction prediction	[39]
Biomedical models				
BioBERT	PubMed, PMC	BERT	Biomedical NER, RE, QA	[59]
PubMedBERT	PubMed	BERT	Biomedical NER, RE, QA	[91]
BioMegatron	PubMed, PMC	Megatron	Biomedical NER, RE, QA	[92]
BioELECTRA	PubMed	ELECTRA	Biomedical NER, RE, QA	[93]
BioALBERT	PubMed, PMC	ALBERT	Biomedical NER, RE, QA	[94]
BioMed‐RoBERTa	PubMed, ChemProt	RoBERTa	Chemical relation classification	[95]
Dr. VAE	Genomics	VAE	Personalized drug response prediction	[96]
MCGAN	text	GANITE	Personalized treatment effect prediction	[97]
S‐VQ‐VAE	Transcriptomics	VAE	Gene expression state prediction	[98]
GP‐GAN	Imaging	GAN	Tumor growth prediction	[99]
ADS‐GAN	Clinical data	GAN	Healthcare data anonymization	[100]
Clinical models				
ClinicalBERT	MIMIC‐III	BERT	Clinical concept extraction	[101]
ClinicalBioBERT	MIMIC‐III	BERT	Biomedical data processing	[102]
UmlsBERT	MIMIC‐III, UMLS	BERT	Clinical data processing	[103]
MedBERT	MIMIC‐III	BERT	Disease prediction	[104]
KM‐BERT	Korean medical literature	BERT	Clinical entity detection	[105]
ClinicalXLNet	MIMIC‐III	XLNet	Clinical entity recognition	[106]
Bio‐GottBERT	German medical corpus	BERT	Medical relation extraction	[107]
Simulation and explainable decision support system models				
LIME	Tabular	Model‐agnostic	Interpret model predictions	[108]
InterpretML	Tabular	Glassbox Models	Interpretable machine learning	[73]
What‐If Tool	Tabular	ML models	Probing ML models	[72]
Model Cards	Tabular	ML models	decision‐making	[109]
DoWhy	Tabular or graph	Causal Inference	Causal inference	[110]

## Applications of advanced DIGITAL twins in personalized medicine

**Fig. 2:**
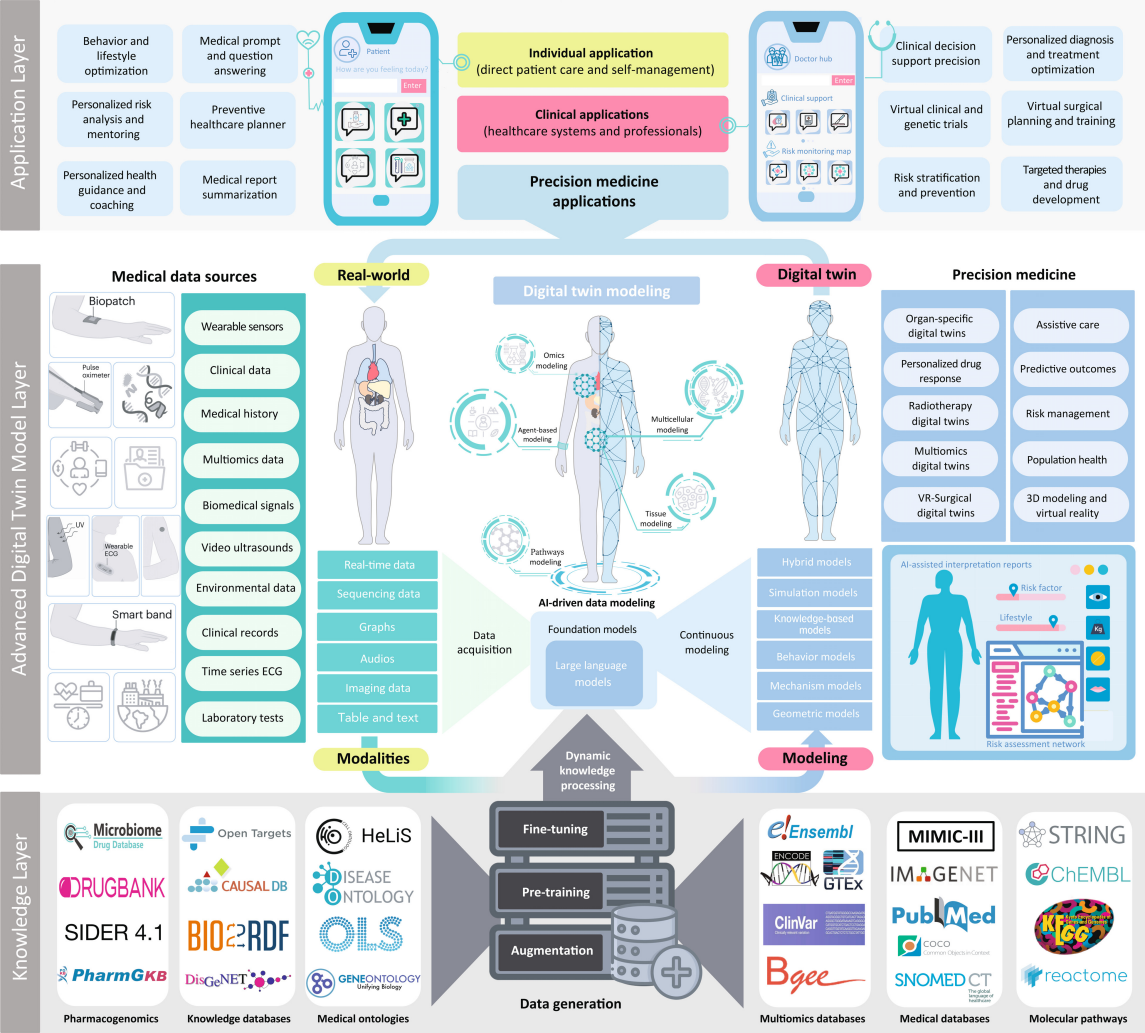
Overview of recent applications of digital twins in medicine: This figure illustrates the architecture of digital twins, highlighting the integration of multimodal data sources (including clinical records, wearable sensors, and multiomics data) with advanced AI‐driven models such as large language models, hybrid models, and simulation models. The figure demonstrates how digital twins are applied across various medical domains, including organ‐specific modeling, personalized drug response, virtual clinical trials, and virtual reality‐surgical twins. The layered structure underscores the role of continuous data acquisition, risk assessment, and precision medicine in optimizing patient care through real‐time feedback loops and clinical decision support.

Advanced MDTs, supported by FMs, have significantly improved multiomics data integration, enabling precise disease modeling and personalized treatment in healthcare [111, 112]. Figure 2 provides an overview of the role of FMs in MDTs and their application in precision medicine.

### Digital twins in preventive medicine

In the field of disease prevention and proactive healthcare management, DTs equipped with FMs offer significant tools for real‐time risk assessment and public health monitoring:

- Population health management: DTs model health dynamics at the population level, predicting trends, assessing risks, and simulating the effects of public health interventions [113]. For instance, during the COVID‐19 pandemic, DTs were deployed to model virus transmission and evaluate intervention strategies, significantly aiding decision‐making processes in public health [114].
- Dynamic risk assessment: DTs continuously update risk profiles as new patient data becomes available, whether from lab tests, wearable devices, or medical records. For example, in cardiovascular care, dynamic assessments of biomarker changes, such as cholesterol or blood pressure, allow clinicians to intervene before critical events such as heart attacks occur [115].
- Real‐time health monitoring: Wearable devices and smart sensors integrate with DTs to provide continuous monitoring of vital health metrics. These real‐time data enable immediate clinical responses, as seen in diabetic patients using glucose monitors, where early interventions prevent hypoglycemic episodes [116].
- Lifestyle optimization: DTs provide personalized lifestyle recommendations by analyzing behavioral data, such as exercise, sleep, and diet. For example, a study on hypertension patients demonstrated how tailored exercise plans and dietary adjustments derived from digital twin simulations improved cardiovascular health outcomes [117].

### Digital twins in disease management and prognosis

DTs have emerged as powerful tools in both chronic disease management and accurate prognosis, combining real‐time data integration, personalized treatment plans, and advanced simulations to improve patient outcomes.

- Chronic disease management: DTs continuously integrate data from genetic, metabolic, and lifestyle factors to manage chronic diseases. In diabetes care, for example, DTs analyze glucose levels, dietary intake, and physical activity to adjust insulin dosages in real‐time, reducing hypoglycemic events by 25% [118]. Cardiovascular disease management has also been enhanced, with DTs optimizing medication regimens based on dynamic patient profiles [119].
- Virtual clinical trials: DTs simulate patient responses to new treatments in virtual clinical trials, reducing reliance on lengthy and expensive traditional trials. A virtual trial of a new antihypertensive drug, for example, reduced the trial's costs by 40% while improving patient selection for phase III trials [17].
- Postsurgical outcome predictions: DTs accurately predict patient recovery trajectories following surgery by integrating real‐time data and past patient outcomes [120]. For instance, in cardiac surgery, DTs predicted risks of infection and graft failures, enabling proactive adjustments to treatment, resulting in 15% faster recovery times [120, 121].
- Enhanced prognostic accuracy: DTs excel in predicting disease progression, particularly in multifactorial diseases. In Alzheimer's disease, for example, DTs were used to predict the rate of cognitive decline with remarkable accuracy, allowing for earlier interventions with disease‐modifying therapies [122].
- Telemedicine and remote care: Telemedicine platforms integrated with DTs allow remote management of chronic diseases [20]. In one study, patients with chronic obstructive pulmonary disease (COPD) in rural areas benefited from remote monitoring and treatment adjustments based on digital twin simulations, reducing hospital admissions by 30% [123].
- Predictive maintenance in medical devices: DTs monitor the performance of implanted medical devices, such as pacemakers or insulin pumps. By simulating device function and predicting failures, DTs enable proactive interventions, reducing device malfunctions and improving patient safety [124].

### Digital twins in pharmacology and medication

Pretrained LLMs are revolutionizing pharmaceutical development, especially in omics data analysis for vaccines, therapeutic target identification, and drug repurposing and discovery, and personalized medication strategies, DTs offer substantial advancements by integrating multiomics data with predictive models:

- Predicting novel drug targets: FMs analyze complex biological data, such as genomic and proteomic profiles, to identify potential drug targets. DTs simulate how targeting specific genes or proteins would impact patient outcomes. In cancer research, for example, DTs were used to simulate the effects of inhibiting certain oncogenes, leading to the identification of novel drug targets [125].
- Omics‐based drug response predictions: By integrating multiomics data, DTs predict individual drug responses, particularly in fields such as oncology and pharmacogenomics. A recent study on chemotherapy for breast cancer patients demonstrated how DTs accurately predicted drug efficacy and toxicity based on genetic and transcriptomic data, improving survival rates by 25% [126].
- Repurposing existing drugs: DTs enable the repurposing of approved drugs for new therapeutic indications. For instance, a digital twin model identified an antihypertensive drug as a promising treatment for chronic kidney disease by simulating shared molecular pathways, accelerating the drug repurposing process. CodonBERT [127] exemplifies this, employing a multi‐head attention transformer to analyze 10 million mRNA sequences, thus advancing mRNA vaccine efficacy by accurately predicting protein synthesis and mRNA stability—key for mRNA‐based therapies [125, 128].
- Antibody design: LLMs have significantly improved the prediction of antigen‐receptor and antigen–antibody interactions. By accurately modeling peptide bindings to HLA molecules, these models treat peptides as ‘linguistic units’ of biology. MHCRoBERTa [129], based on the BERT framework, specializes in pMHC‐I predictions by analyzing amino acid sequences to differentiate alleles precisely, while BERTMHC [130] enhances pMHC‐II‐binding predictions using a dataset of 2413 MHC–peptide pairs across 47 alleles, addressing key gaps in this area. Additionally, models like AbLang [131] and AntiBERTa [132] correct sequencing errors and analyze antibody sequences with superior accuracy, while EATLM utilizes stacked transformers with specialized pretraining tasks, collectively advancing bioinformatics and therapeutic development in immunotherapy.

## Challenges and limitations

Although FMs hold transformative potential for multiomics data analysis and digital twin systems, several challenges impede their full implementation and scalability in healthcare. As summarized in Table 2, addressing these limitations is crucial for FMs to fulfill their promise in precision medicine.

- Data availability and quality: The effectiveness of FMs heavily relies on the availability of large, high‐quality datasets for pretraining and fine‐tuning. In the multiomics field, datasets are often limited in size, contain missing data, or exhibit noise, which impairs model performance. This limitation also affects the generalizability of FMs, particularly across diverse patient populations. Ensuring access to comprehensive and diverse datasets is critical for training robust models.
- Computational demands: The high computational power required to train and deploy FMs, especially in multiomics applications, remains a major barrier. Processing large‐scale data streams from genomics, proteomics, and other omics requires scalable high‐performance computing infrastructure, which is not always accessible to healthcare institutions. This also limits the ability to perform real‐time updates in DTs.
- Interpretability and trust in AI systems: Many FMs function as black boxes, providing little to no transparency in how predictions are made. This lack of interpretability poses a significant challenge for clinicians who need to trust and understand AI‐driven predictions before integrating them into clinical workflows. Without clear explanations of model decisions, clinical adoption remains limited.
- Causal relationships and biological insight: Current FMs struggle to capture the complex causal relationships within multiomics data that are crucial for accurate predictions of disease progression and treatment outcomes. The absence of causal inference limits the biological interpretability of model predictions, making it harder to generate meaningful insights for precision medicine.
- Ethical and privacy concerns: The large datasets required for training FMs often include sensitive patient information, raising concerns about privacy and data security. Ensuring compliance with healthcare regulations, such as HIPAA and GDPR, is crucial to maintaining patient trust. Moreover, biased data can lead to biased models, perpetuating inequalities in healthcare delivery. Addressing these issues is fundamental for the responsible use of FMs in clinical settings.

**Table 2:** Summary of main limitations and recommendations for foundation models in digital twin systems for medicine and bioinformatics.

Key limitations	Recommended solutions
Limited generalizability across patient populations	Use diverse, population‐specific datasets to improve model adaptability and robustness
Lack of real‐time data integration	Establish infrastructure that supports continuous, real‐time data flows for accurate digital twin updates
Challenges in multimodal data integration	Develop advanced models to integrate data across multiomics, imaging, and clinical records, enabling a complete patient profile
Interpretability challenges with complex models	Improve transparency with explainable AI techniques to ensure clinician trust and usability
High computational requirements	Optimize models and use scalable cloud resources to meet computational demands efficiently
Limited validation in clinical settings	Enhance model robustness by testing and validating in varied, real‐world clinical environments
Dependency on high‐quality labeled data	Utilize semi‐supervised learning and generate synthetic data to reduce reliance on extensive labeled datasets
Inadequate modeling of rare diseases	Apply transfer learning to enhance model accuracy in predicting rare disease outcomes
Bias in training data	Ensure training with diverse datasets to minimize bias and improve equitable predictions
Difficulty in capturing causal relationships	Integrate causal inference models to strengthen biological insights and prediction accuracy
Data privacy and security concerns	Implement privacy‐preserving methods, such as federated learning, to protect patient data
Overfitting in models trained on specific diseases	Use regularization and robust cross‐validation techniques to mitigate overfitting risks
Inability to effectively analyze temporal data	Enhance models with time‐series analysis capabilities crucial for real‐time applications
Challenges in validating treatment response predictions	Use reinforcement learning to refine treatment simulations and improve response validation
Slow adaptation to evolving medical knowledge	Incorporate continuous learning mechanisms to keep models updated with the latest medical data

## Future directions

Addressing the challenges highlighted in the previous section is critical to advancing the application of FMs in precision medicine. Future directions for improving the capabilities of FMs and overcoming current limitations include:

- Real‐time multiomics integration: As wearable biosensors, medical imaging, and other real‐time data sources continue to evolve, integrating continuous data streams from multiomics profiles will be essential. Future FMs must be capable of processing real‐time data from genomics, proteomics, metabolomics, and clinical sensors to enable dynamic disease monitoring and personalized treatment adjustments. Advances in computational infrastructure are necessary to support these real‐time, multi‐stream integrations efficiently.
- Enhancing interpretability and explainability: Future FMs must prioritize interpretability to gain clinical trust and widespread adoption. Explainable AI techniques should be incorporated to clarify the rationale behind predictions. These models should not only be able to provide accurate results but also offer insights into the underlying biological mechanisms that drive disease progression. Interactive dashboards that allow clinicians to visualize predictions and explore the reasoning behind them will be essential in enhancing usability.
- Capturing causal relationships and reducing bias: Integrating causal inference techniques with FMs will be critical for producing biologically meaningful predictions. Capturing the causal pathways between multiomics data and clinical outcomes will enable more reliable disease progression models and treatment forecasts. Additionally, efforts must be made to address biases inherent in training data by ensuring that FMs are built on diverse and representative datasets. Bias mitigation strategies, including fairness‐aware learning algorithms, will help ensure equitable healthcare recommendations across populations.
- Ethical data sharing and privacy preservation: The creation of collaborative ecosystems where healthcare institutions and MDTs platforms can securely share data will be fundamental for progress. Privacy‐preserving techniques such as federated learning will play a crucial role in enabling this data exchange while protecting patient confidentiality. These frameworks will ensure compliance with regulations and reduce the risks of data breaches, paving the way for wider data access without compromising privacy.
- Continuous learning and model adaptation: In a rapidly evolving medical landscape, FMs must be equipped with continuous learning capabilities. This will enable FMs to incorporate new medical knowledge, clinical guidelines, and research findings dynamically. By staying updated with the latest advancements in genomics, therapeutics, and diagnostics, FMs can maintain their relevance and accuracy in personalized healthcare applications.

In conclusion, while FMs have opened new frontiers in personalized medicine and multiomics analysis, their full potential will only be realized by addressing the current challenges and implementing forward‐looking solutions. By fostering collaboration across clinical and computational disciplines, enhancing model transparency, and ensuring equitable, privacy‐conscious use of patient data, FMs will continue to transform the future of healthcare.

## Conflict of interest

The authors declare no conflict of interest.

## Author contributions

The study was designed by SA and TG. The literature search was conducted and data collection by SA, figures are designed by SA and validated by TG and XG, the methodology by SA, and supervision by TG. SA drafted the manuscript with assistance from TG. SA and TG directly accessed and verified the underlying data reported in the manuscript. XG revised the manuscript. All authors were involved in the study design and data interpretation. All authors approved the final version of the manuscript, had full access to all the data in the study, and had final responsibility for the decision to submit for publication.

## References

[1] Sun T , He X and Li Z (2023) Digital twin in healthcare: recent updates and challenges. Digit Health 9, 20552076221149651. 36636729 10.1177/20552076221149651 PMC9830576

[2] Rosen R , Von Wichert G , Lo G and Bettenhausen KD (2015) About the importance of autonomy and digital twins for the future of manufacturing. IFAC Pap OnLine 48, 567–572.

[3] Kamel Boulos MN and Zhang P (2021) Digital twins: from personalized medicine to precision public health. J Pers Med 11, 745. 34442389 10.3390/jpm11080745 PMC8401029

[4] Cellina M , Cè M , Alì M , Irmici G , Ibba S , Caloro E , Fazzini D , Oliva G and Papa S (2023) Digital twins: the new frontier for personalized medicine. Appl Sci 13, 7940.

[5] Geanta M , Tanwar AS , Lehrach H , Satyamoorthy K and Brand A (2022) Horizon scanning: rise of planetary health genomics and digital twins for pandemic preparedness. OMICS 26, 93–100. 34851750 10.1089/omi.2021.0062

[6] Weifei H , Zhang T , Deng X , Liu Z and Tan J (2021) Digital twin: a state‐of‐the‐art review of its enabling technologies, applications and challenges. J Intell Manuf Spec Equip 2, 1–34.

[7] Peirlinck M , Sahli Costabal F , Yao J , Guccione JM , Tripathy S , Wang Y , Ozturk D , Segars P , Morrison TM , Levine S *et al*. (2021) Precision medicine in human heart modeling: perspectives, challenges, and opportunities. Biomech Model Mechanobiol 20, 803–831. 33580313 10.1007/s10237-021-01421-z PMC8154814

[8] Ghebrehiwet I , Zaki N , Damseh R and Mohamad MS (2024) Revolutionizing personalized medicine with generative ai: a systematic review. Artif Intell Rev 57, 1–41.

[9] Khan W , Leem S , See KB , Wong JK , Zhang S and Fang R (2024) A comprehensive survey of foundation models in medicine. *arXiv* preprint arXiv:2406.10729. 10.48550/arXiv.2406.10729 [PREPRINT] 40031197

[10] Hossain SMM , Saha SK , Banik S and Banik T (2023) A new era of mobility: exploring digital twin applications in autonomous vehicular systems. In *2023 IEEE World AI IoT Congress (AIIoT)*, pp. 493–499. IEEE.

[11] Hall EC (1996) Journey to the Moon: The History of the Apollo Guidance Computer. AIAA, Reston, VA.

[12] LeBlanc MB (2020) Digital twin technology for enhanced upstream capability in oil and gas. PhD thesis, Massachusetts Institute of Technology.

[13] Gelernter D (1993) Mirror Worlds: Or the Day Software Puts the Universe in a Shoebox. How it Will Happen and What it Will Mean. Oxford University Press, Oxford, UK.

[14] Li L , Lei B and Mao C (2022) Digital twin in smart manufacturing. J Ind Inf Integr 26, 100289.

[15] Armeni P , Polat I , De Rossi LM , Di‐aferia L , Meregalli S and Gatti A (2022) Digital twins in healthcare: is it the beginning of a new era of evidence‐based medicine? A critical review. J Pers Med 12, 1255. 36013204 10.3390/jpm12081255 PMC9410074

[16] Vallée A (2023) Digital twin for healthcare systems. Front Digit Health 5, 1253050. 37744683 10.3389/fdgth.2023.1253050 PMC10513171

[17] Wang Y , Fu T , Xu Y , Ma Z , Xu H , Du B , Lu Y , Gao H , Wu J and Chen J (2024) Twin‐gpt: digital twins for clinical trials via large language model. ACM Trans Multimed Comput Commun Appl

[18] Katsoulakis E , Wang Q , Huanmei W , Shahriyari L , Fletcher R , Liu J , Achenie L , Liu H , Jackson P , Xiao Y *et al*. (2024) Digital twins for health: a scoping review. NP J Digit Med 7, 77. 10.1038/s41746-024-01073-0 PMC10960047 38519626

[19] Dihan MS , Akash AI , Tasneem Z , Das P , Das SK , Islam MR , Islam MM , Badal FR , Ali MF , Ahmed MH *et al*. (2024) Digital twin: data exploration, architecture, implementation and future. Heliyon 10, e26503. 38444502 10.1016/j.heliyon.2024.e26503 PMC10912257

[20] Haleem A , Javaid M , Singh RP and Suman R (2023) Exploring the revolution in healthcare systems through the applications of digital twin technology. Biomed Tech 4, 28–38.

[21] Moor M , Banerjee O , Abad ZSH , Krumholz HM , Leskovec J , Topol EJ and Rajpurkar P (2023) Foundation models for generalist medical artificial intelligence. Nature 616, 259–265. 37045921 10.1038/s41586-023-05881-4

[22] Vaswani A (2017) Attention is all you need. Adv Neural Inf Process Syst

[23] Yenduri G , Ramalingam M , Selvi GC , Supriya Y , Srivastava G , Maddikunta PKR , Raj GD , Jhaveri RH , Prabadevi B , Wang W *et al*. (2024) Gpt (generative pre‐trained transformer)–a comprehensive review on enabling technologies, potential applications, emerging challenges, and future directions. IEEE Access 12, 54608–54649.

[24] Devlin J (2018) Bert: pre‐training of deep bidirectional transformers for language understanding. *arXiv* preprint arXiv:1810.04805. 10.48550/arXiv.1810.04805 [PREPRINT]

[25] Joschka Haltaufderheide and Robert Ranisch (2024) The ethics of chatgpt in medicine and healthcare: a systematic review on large language models (llms). NP J Digit Med 7, 183. 10.1038/s41746-024-01157-x PMC11231310 38977771

[26] AlSaad R , Abd‐Alrazaq A , Boughorbel S , Ahmed A , Renault M‐A , Damseh R and Sheikh J (2024) Multi‐modal large language models in health care: applications, challenges, and future outlook. J Med Internet Res 26, e59505. 39321458 10.2196/59505 PMC11464944

[27] Singhal P , Walambe R , Ramanna S and Kotecha K (2023) Domain adaptation: challenges, methods, datasets, and applications. IEEE Access 11, 6973–7020.

[28] Jahan I , Laskar MTR , Peng C and Huang JX (2024) A comprehensive evaluation of large language models on benchmark biomedical text processing tasks. Comput Biol Med 171, 108189. 38447502 10.1016/j.compbiomed.2024.108189

[29] Ross TD and Gopinath A (2024) Chaining thoughts and llms to learn dna structural biophysics. *arXiv* preprint arXiv:2403.01332. 10.48550/arXiv.2403.01332 [PREPRINT]

[30] Chatterjee S , Bhattacharya M , Lee S‐S and Chakraborty C (2023) Can artificial intelligence‐strengthened chatgpt or other large language models transform nucleic acid research? Mol Ther Nucleic Acids 33, 205–207. 37727444 10.1016/j.omtn.2023.06.019 PMC10505907

[31] Lam HYI , Ong XE and Mutwil M (2024) Large language models in plant biology. Trends Plant Sci 29, 1145–1155. 38797656 10.1016/j.tplants.2024.04.013

[32] Zhang D , Zhang W , He B , Zhang J , Qin C and Yao J (2023) Dnagpt: a generalized pretrained tool for multiple dna sequence analysis tasks. *bioRxiv* 2023‐07. 10.1101/2023.07.11.548628 [PREPRINT]

[33] Claringbould A and Zaugg JB (2021) Enhancers in disease: molecular basis and emerging treatment strategies. Trends Mol Med 27, 1060–1073. 34420874 10.1016/j.molmed.2021.07.012

[34] Ji Y , Zhou Z , Liu H and Davuluri RV (2021) Dnabert: pre‐trained bidirectional encoder representations from transformers model for dna‐language in genome. Bioinformatics 37, 2112–2120. 33538820 10.1093/bioinformatics/btab083 PMC11025658

[35] Zhou Z , Ji Y , Li W , Dutta P , Davuluri R and Liu H (2023) Dnabert‐2: efficient foundation model and benchmark for multi‐species genome. *arXiv* preprint arXiv:2306.15006. 10.48550/arXiv.2306.15006 [PREPRINT]

[36] Li J , Zhourun W , Lin W , Luo J , Zhang J , Chen Q‐C and Chen J (2023) iEnhancer‐ELM: improve enhancer identification by extracting position‐related multiscale contextual information based on enhancer language models. Bioinform Adv 3, vbad043. 37113248 10.1093/bioadv/vbad043 PMC10125906

[37] Le NQK , Ho Q‐T , Nguyen V‐N and Chang J‐S (2022) Bert‐promoter: an improved sequence‐based predictor of dna promoter using bert pre‐trained model and shap feature selection. Comput Biol Chem 99, 107732. 35863177 10.1016/j.compbiolchem.2022.107732

[38] Sanabria M , Hirsch J and Poetsch AR (2023) The human genome's vocabulary as proposed by the dna language model grover. *bioRxiv* 2023‐07. 10.1101/2023.07.19.549677 [PREPRINT]

[39] Luo H , Shan W , Chen C , Ding P and Luo L (2023) Improving language model of human genome for dna–protein binding prediction based on task‐specific pre‐training. Interdiscip Sci 15, 32–43. 36136096 10.1007/s12539-022-00537-9

[40] Zhang Y , Lang M , Jiang J , Gao Z , Fan X , Litfin T , Chen K , Singh J , Huang X , Song G *et al*. (2024) multiple sequence alignment‐based rna language model and its application to structural inference. Nucleic Acids Res 52, e3. 37941140 10.1093/nar/gkad1031 PMC10783488

[41] Chen J , Hu Z , Sun S , Tan Q , Wang Y , Yu Q , Zong L , Hong L , Xiao J , Shen T *et al*. (2022) Interpretable RNA foundation model from unannotated data for highly accurate RNA structure and function predictions. *arXiv* preprint arXiv:2204.00300. 10.48550/arXiv.2204.00300 [PREPRINT]

[42] Kalicki CH and Haritaoglu ED (2020) RNAbert: RNA family classification and secondary structure prediction with BERT pretrained on RNA sequences. https://cs230.stanford.edu/projects_fall_2022/reports/88.pdf

[43] Chen K , Zhou Y , Ding M , Wang Y , Ren Z and Yang Y (2023) Self‐supervised learning on millions of premrna sequences improves sequence‐based rna splicing prediction. *bioRxiv* 2023‐01. 10.1101/2023.01.31.526427 [PREPRINT] PMC11009468 38605640

[44] Shen H , Liu J , Jiani H , Shen X , Zhang C , Dan W , Feng M , Yang M , Li Y , Yang Y *et al*. (2023) Generative pretraining from large‐scale transcriptomes for single‐cell deciphering. iScience 26, 106536. 37187700 10.1016/j.isci.2023.106536 PMC10176267

[45] Chen Y and Zou J (2023) Genept: a simple but effective foundation model for genes and cells built from chatgpt. *bioRxiv*. 10.1101/2023.10.16.562533 [PREPRINT]

[46] Zhang L , Qin X , Liu M , Liu G and Ren Y (2021) Bert‐m7g: a transformer architecture based on bert and stacking ensemble to identify rna n7‐methylguanosine sites from sequence information. Comput Math Methods Med 2021, 7764764. 34484416 10.1155/2021/7764764 PMC8413034

[47] Soylu NN and Sefer E (2023) Bert2ome: prediction of 2′‐o‐methylation modifications from rna sequence by transformer architecture based on bert. IEEE/ACM Trans Comput Biol Bioinform 20, 2177–2189. 37819796 10.1109/TCBB.2023.3237769

[48] Rao R , Bhattacharya N , Thomas N , Duan Y , Chen P , Canny J , Abbeel P and Song Y (2019) Evaluating protein transfer learning with tape. Adv Neural Inf Process Syst 32, 9689–9701. 33390682 PMC7774645

[49] Elnaggar A , Heinzinger M , Dallago C , Ghalia Rehawi Y , Wang LJ , Gibbs T , Feher T , Angerer C , Steinegger M *et al*. (2021) Prottrans: toward understanding the language of life through self‐supervised learning. IEEE Trans Pattern Anal Mach Intell 44, 7112–7127. 10.1109/TPAMI.2021.3095381 34232869

[50] Strodthoff N , Wagner P , Wenzel M and Samek W (2020) Udsmprot: universal deep sequence models for protein classification. Bioinformatics 36, 2401–2409. 31913448 10.1093/bioinformatics/btaa003 PMC7178389

[51] Mrázek J and Kypr J (1993) Unirep: a microcomputer program to find unique and repetitive nucleotide sequences in genomes. Bioinformatics 9, 355–360. 10.1093/bioinformatics/9.3.355 8391893

[52] Sargsyan K and Lim C (2024) Using protein language models for protein interaction hot spot prediction with limited data. BMC Bioinformatics 25, 115. 38493120 10.1186/s12859-024-05737-2 PMC10943781

[53] Wang Z , Zhang Q , HU S‐W , Yu H , Jin X , Gong Z and Chen H (2022) Multi‐level protein structure pre‐training via prompt learning. In *The Eleventh International Conference on Learning Representations*.

[54] Brandes N , Ofer D , Peleg Y , Rappoport N and Linial M (2022) Proteinbert: a universal deep‐learning model of protein sequence and function. Bioinformatics 38, 2102–2110. 35020807 10.1093/bioinformatics/btac020 PMC9386727

[55] Sho T , Hasan MM , Deng H‐W and Kurata H (2022) Bert6ma: prediction of dna n6‐methyladenine site using deep learning‐based approaches. Brief Bioinform 23, bbac053. 35225328 10.1093/bib/bbac053 PMC8921755

[56] Yingying Y , He W , Jin J , Xiao G , Cui L , Zeng R and Wei L (2021) Idna‐abt: advanced deep learning model for detecting dna methylation with adaptive features and transudative information maximization. Bioinformatics 37, 4603–4610. 34601568 10.1093/bioinformatics/btab677

[57] Zeng W , Gautam A and Huson DH (2023) Mulan‐ methyl—multiple transformer‐based language models for accurate dna methylation prediction. Gigascience 12, giad054. 10.1093/gigascience/giad054 PMC10367125 37489753

[58] Feng H , Wang S , Wang Y , Ni X , Yang Z , Hu X‐m and Yang S (2023) Lnccat: an orf attention model to identify lncrna based on ensemble learning strategy and fused sequence information. Comput Struct Biotechnol J 21, 1433–1447. 36824229 10.1016/j.csbj.2023.02.012 PMC9941877

[59] Lee J , Yoon W , Kim S , Kim D , Kim S , So CH and Kang J (2020) Biobert: a pre‐trained biomedical language representation model for biomedical text mining. Bioinformatics 36, 1234–1240. 31501885 10.1093/bioinformatics/btz682 PMC7703786

[60] Han Q , Tian S and Zhang J (2021) A pubmedbert‐based classifier with data augmentation strategy for detecting medication mentions in tweets. *arXiv* preprint arXiv:2112.02998. 10.48550/arXiv.2112.02998 [PREPRINT]

[61] Singhal K , Azizi S , Tao T , Sara Mahdavi S , Wei J , Chung HW , Scales N , Tanwani A , Lewis HC , Pfohl S *et al*. (2023) Large language models encode clinical knowledge. Nature 620, 172–180. 37438534 10.1038/s41586-023-06291-2 PMC10396962

[62] Huang K , Altosaar J and Ranganath R (2019) Clinicalbert: Modeling clinical notes and predicting hospital readmission. *arXiv* preprint arXiv:1904.05342. 10.48550/arXiv.1904.05342 [PREPRINT]

[63] Michalopoulos G , Wang Y , Kaka H , Chen H and Wong A (2020) Umlsbert: clinical domain knowledge augmentation of contextual embeddings using the unified medical language system metathesaurus. *arXiv* preprint arXiv:2010.10391. 10.48550/arXiv.2010.10391 [PREPRINT]

[64] Mannion A , Chevalier T , Schwab D and Geouriot L (2023) Umls‐kgi‐bert: data‐centric knowledge integration in transformers for biomedical entity recognition. *arXiv* preprint arXiv:2307.11170. 10.48550/arXiv.2307.11170 [PREPRINT]

[65] Rampášek L , Hidru D , Smirnov P , Kains BH and Goldenberg A (2019) Dr. vae: improving drug response prediction via modeling of drug perturbation effects. Bioinformatics 35, 3743–3751. 30850846 10.1093/bioinformatics/btz158 PMC6761940

[66] Han C , Kitamura Y , Kudo A , Ichinose A , Rundo L , Furukawa Y , Umemoto K , Li Y and Nakayama H (2019) Synthesizing diverse lung nodules wherever massively: 3d multi‐conditional gan‐based ct image augmentation for object detection. In *2019 International Conference on 3D Vision (3DV)*, pp. 729–737. IEEE.

[67] Basori AH , Malebary SJ and Alesawi S (2023) Hybrid deep convolutional generative adversarial network (dcgan) and xtreme gradient boost for x‐ray image augmentation and detection. Appl Sci 13, 12725.

[68] Linardatos P , Papastefanopoulos V and Kotsiantis S (2020) Explainable ai: a review of machine learning interpretability methods. Entropy 23, 18. 33375658 10.3390/e23010018 PMC7824368

[69] Lan Y , Li Z and Lin W (2024) “Why should i trust you?”: exploring interpretability in machine learning approaches for indirect shm. eJ Nondestruct Test Ultrason 2024,

[70] Ribeiro MT , Singh S and Guestrin C (2016) “why should i trust you?” explaining the predictions of any classifier. In *Proceedings of the 22nd ACM SIGKDD international conference on knowledge discovery and data mining*, pp. 1135–1144.

[71] Lundberg SM , Erion GG and Lee S‐I (2018) Consistent individualized feature attribution for tree ensembles. *arXiv* preprint arXiv:1802.03888. 10.48550/arXiv.1802.03888 [PREPRINT]

[72] Wexler J , Pushkarna M , Bolukbasi T , Wattenberg M , Viégas F and Wilson J (2019) The what‐if tool: interactive probing of machine learning models. IEEE Trans Vis Comput Graph 26, 56–65. 31442996 10.1109/TVCG.2019.2934619

[73] Nori H , Jenkins S , Koch P and Caruana R (2019) Interpretml: a unified framework for machine learning interpretability. *arXiv* preprint arXiv:1909.09223. 10.17148/IJARCCE.2023.125225 [PREPRINT]

[74] Schneider L , Bischl B and Thomas J (2023) Multi‐objective optimization of performance and interpretability of tabular supervised machine learning models. In *Proceedings of the genetic and evolutionary computation conference*, pp. 538–547.

[75] Baniya B (2024) Integrating and validating heart health calculators in medical platforms. Master's thesis, UiT Norges arktiske universitet.

[76] Liao L , Feng M and Yang M (2024) Human guided cross‐modal reasoning with semantic attention learning for visual question answering. In *ICASSP 2024–2024 IEEE International Conference on Acoustics, Speech and Signal Processing (ICASSP)*, pp. 2775–2779. IEEE.

[77] Dalla‐Torre H , Gonzalez L , Mendoza‐Revilla J , Lopez Carranza N , Grzywaczewski AH , Oteri F , Dallago C , Trop E , de Almeida BP , Sirelkhatim H *et al*. (2023) The nucleotide transformer: building and evaluating robust foundation models for human genomics. *bioRxiv* 2023‐01. 10.1101/2023.01.11.523679 [PREPRINT] PMC11810778 39609566

[78] Luo H , Chen C , Shan W , Ding P and Luo L (2022) ienhancer‐bert: a novel transfer learning architecture based on dna‐language model for identifying enhancers and their strength. In *International Conference on Intelligent Computing*, pp. 153–165. Springer.

[79] Benegas G , Batra SS and Song YS (2023) Dna language models are powerful predictors of genome‐wide variant effects. Proc Natl Acad Sci USA 120, e2311219120. 37883436 10.1073/pnas.2311219120 PMC10622914

[80] Li G , Yao S and Fan L (2024) Prostage: predicting effects of mutations on protein stability by using protein embeddings and graph convolutional networks. J Chem Inf Model 64, 340–347. 38166383 10.1021/acs.jcim.3c01697 PMC10806799

[81] Brandes N , Ofer D , Peleg Y , Rappoport N and Linial M (2022) ProteinBERT: a universal deep‐learning model of protein sequence and function. Bioinformatics 38 (8), 2102–2110. 35020807 10.1093/bioinformatics/btac020 PMC9386727

[82] Le VT , Zhan ZJ , Malik MS and Ou YY (2024) ProtTrans and multi‐window scanning convolutional neural networks for the prediction of protein‐peptide interaction sites. J Mol Graph Model 130, 108777. 38642500 10.1016/j.jmgm.2024.108777

[83] Jumper J , Evans R , Pritzel A , Green T , Figurnov M , Ronneberger O , Tunyasuvunakool K , Bates R , Žídek A , Potapenko A *et al*. (2021) Highly accurate protein structure prediction with AlphaFold. Nature 596, 583–589. 34265844 10.1038/s41586-021-03819-2 PMC8371605

[84] Meier J , Rao R , Verkuil R , Liu J , Sercu T and Rives A (2021) Language models enable zero‐shot prediction of the effects of mutations on protein function. Adv Neural Inf Proces Syst 34, 29287–29303.

[85] Rao RM , Liu J , Verkuil R , Meier J , Canny J , Abbeel P , Sercu T and Rives A (2021) MSA transformer. In International Conference on Machine Learningpp. 8844–8856. PMLR,

[86] Ferruz N , Schmidt S and Höcker B (2022) ProtGPT2 is a deep unsupervised language model for protein design. Nat Commun 13 (1), 4348. 35896542 10.1038/s41467-022-32007-7 PMC9329459

[87] Teufel F , Almagro Armenteros JJ , Johansen AR , Gíslason MH , Pihl SI , Tsirigos KD , Winther O , Brunak S , von Heijne G and Nielsen H (2022) SignalP 6.0 predicts all five types of signal peptides using protein language models. Nat Biotechnol 40, 1023–1025. 34980915 10.1038/s41587-021-01156-3 PMC9287161

[88] Notin P , Dias M , Frazer J , Marchena‐Hurtado J , Gomez AN , Marks D and Gal Y (2022) Tranception: protein fitness prediction with autoregressive transformers and inference‐time retrieval. In International Conference on Machine Learningpp. 16990–17017. PMLR,

[89] Bernhofer M and Rost B (2022) TMbed: transmembrane proteins predicted through language model embeddings. BMC Bioinformatics 23 (1), 326. 35941534 10.1186/s12859-022-04873-x PMC9358067

[90] Jin M , Xue H , Wang Z , Kang B , Ye R , Zhou K , Du M and Zhang Y (2024) ProLLM: protein chain‐of‐thoughts enhanced LLM for protein–protein interaction prediction. bioRxiv 2024‐04. doi: 10.48550/arXiv.2405.06649

[91] Gu Y , Tinn R , Cheng H , Lucas M , Usuyama N , Liu X , Naumann T , Gao J and Poon H (2021) Domain‐specific language model pretraining for biomedical natural language processing. ACM Trans Comput Heal 3 (1), 1–23.

[92] Shin HC , Zhang Y , Bakhturina E , Puri R , Patwary M , Shoeybi M and Mani R (2020) BioMegatron: larger biomedical domain language model. arXiv preprint arXiv:2010.06060 doi: 10.48550/arXiv.2010.06060

[93] Kanakarajan KR , Kundumani B and Sankarasubbu M (2021) BioELECTRA: pretrained biomedical text encoder using discriminators. In Proceedings of the 20th Workshop on Biomedical Language Processingpp. 143–154. Association for Computational Linguistics,

[94] Naseem U , Khushi M , Reddy V , Rajendran S , Razzak I and Kim J (2021) Bioalbert: A simple and effective pre‐trained language model for biomedical named entity recognition. In 2021 International Joint Conference on Neural Networks (IJCNN)pp. 1–7. IEEE,

[95] Lewis P , Ott M , Du J and Stoyanov V (2020) Pretrained language models for biomedical and clinical tasks: understanding and extending the state‐of‐the‐art. In Proceedings of the 3rd Clinical Natural Language Processing Workshoppp. 146–157. Association for Computational Linguistics,

[96] Rampášek L , Hidru D , Smirnov P , Haibe‐Kains B and Goldenberg A (2019) Dr. VAE: improving drug response prediction via modeling of drug perturbation effects. Bioinformatics 35, 3743–3751. 30850846 10.1093/bioinformatics/btz158 PMC6761940

[97] Mroueh Y , Sercu T and Goel V (2017) Mcgan: Mean and covariance feature matching gan. In International Conference on Machine Learningpp. 2527–2535. PMLR,

[98] Xue Y , Ding MQ and Lu X (2020) Learning to encode cellular responses to systematic perturbations with deep generative models. NPJ Syst Biol Appl 6 (1), 35. 33159077 10.1038/s41540-020-00158-2 PMC7648057

[99] Elazab A , Wang C , Gardezi SJS , Bai H , Hu Q , Wang T , Chang C and Lei B (2020) GP‐GAN: Brain tumor growth prediction using stacked 3D generative adversarial networks from longitudinal MR Images. Neural Netw 132, 321–332. 32977277 10.1016/j.neunet.2020.09.004

[100] Yoon J , Drumright LN and Van Der Schaar M (2020) Anonymization through data synthesis using generative adversarial networks (ADS‐GAN). IEEE J Biomed Health Inform 24, 2378–2388. 32167919 10.1109/JBHI.2020.2980262

[101] Huang K , Altosaar J and Ranganath R (2019) Clinicalbert: Modeling clinical notes and predicting hospital readmission. arXiv preprint arXiv:1904.05342 doi: 10.48550/arXiv.1904.05342

[102] Alsentzer E , Murphy JR , Boag W , Weng WH , Jin D , Naumann T and McDermott M (2019) Publicly available clinical BERT embeddings. arXiv preprint arXiv:1904.03323

[103] Michalopoulos G , Wang Y , Kaka H , Chen H and Wong A (2020) Umlsbert: Clinical domain knowledge augmentation of contextual embeddings using the unified medical language system metathesaurus. arXiv preprint arXiv:2010.10391

[104] Rasmy L , Xiang Y , Xie Z , Tao C and Zhi D (2021) Med‐BERT: pretrained contextualized embeddings on large‐scale structured electronic health records for disease prediction. NPJ Digit Med 4 (1), 86. 34017034 10.1038/s41746-021-00455-y PMC8137882

[105] Kim Y , Kim JH , Lee JM , Jang MJ , Yum YJ , Kim S , Shin U , Kim YM , Joo HJ and Song S (2022) A pre‐trained BERT for Korean medical natural language processing. Sci Rep 12 (1), 13847. 35974113 10.1038/s41598-022-17806-8 PMC9381714

[106] Huang K , Singh A , Chen S , Moseley ET , Deng CY , George N and Lindvall C (2019) Clinical XLNet: Modeling sequential clinical notes and predicting prolonged mechanical ventilation. arXiv preprint arXiv:1912.11975

[107] Lentzen M , Madan S , Lage‐Rupprecht V , Kühnel L , Fluck J , Jacobs M , Mittermaier M , Witzenrath M , Brunecker P , Hofmann‐Apitius M *et al*. (2022) Critical assessment of transformer‐based AI models for German clinical notes. JAMIA open 5, ooac087. 36380848 10.1093/jamiaopen/ooac087 PMC9663939

[108] Bramhall S , Horn H , Tieu M and Lohia N (2020) Qlime‐a quadratic local interpretable model‐agnostic explanation approach. SMU Data Sci Rev 3, 4.

[109] Mitchell M , Wu S , Zaldivar A , Barnes P , Vasserman L , Hutchinson B , Spitzer E , Raji ID and Gebru T (2019) Model cards for model reporting. In Proceedings of the conference on fairness, accountability, and transparencypp. 220–229.

[110] Sharma A and Kiciman E (2020) DoWhy: An end‐to‐end library for causal inference. arXiv preprint arXiv:2011.04216

[111] Labarga A , Martínez‐Gonzalez J and Barajas M (2024) Integrative multi‐omics analysis for etiology classification and biomarker discovery in stroke: advancing towards precision medicine. Biology 13, 338. 38785820 10.3390/biology13050338 PMC11149453

[112] Sakhaa Alsaedi , Mineta K , Tamura N , Gao X , Gojobori T and Ogasawara M (2024) Integrative multiomics network analysis of genetic risk factors to infer biomarkers and therapeutic targets for rheumatoid arthritis. 10.21203/rs.3.rs-3607429/v1 PMC12370121 40839671

[113] Alsaedi SB , Mineta K , Gao X and Gojobori T (2023) Computational network analysis of host genetic risk variants of severe covid‐19. Hum Genomics 17, 17. 36859360 10.1186/s40246-023-00454-y PMC9977643

[114] Ren Z , Zhang X and Zhang Z (2021) New evidence on covid‐19 and firm performance. Econ Anal Policy 72, 213–225. 34934261 10.1016/j.eap.2021.08.002 PMC8682735

[115] Mulder ST , Omidvari A‐H , Rueten‐ Budde AJ , P‐HH , Kim K‐H , Bais B , Rousian M , Hai R , Akgun C , van Lennep JR *et al*. (2022) Dynamic digital twin: diagnosis, treatment, prediction, and prevention of disease during the life course. J Med Internet Res 24, e35675. 36103220 10.2196/35675 PMC9520391

[116] Reddy N , Verma N and Dungan K (2023) Monitoring technologies‐continuous glucose monitoring, mobile technology, biomarkers of glycemic control. In Endotext ( Feingold KR , Anawalt B , Blackman MR , Boyce A , Chrousos G , Corpas E , de Herder WW , Dhatariya K , Dungan K , Hofland J , *et al*., eds), MDText.com, Inc., South Dartmouth, MA. 25905275

[117] Ambroselli D , Masciulli F , Romano E , Catanzaro G , Besharat ZM , Massari MC , Ferretti E , Migliaccio S , Izzo L , Ritieni A *et al*. (2023) New advances in metabolic syndrome, from prevention to treatment: the role of diet and food. Nutrients 15, 640. 36771347 10.3390/nu15030640 PMC9921449

[118] Holloway D , James S , Ekinci E and Craft J (2023) Systematic review of the effectiveness of nurse‐led care in reducing glycated haemoglobin in adults with type 1 or 2 diabetes. Int J Nurs Pract 29, e13135. 36733216 10.1111/ijn.13135

[119] Qiu J , Han W , Zhu J , Xu M , Rosenberg M , Liu E , Weber D and Zhao D (2023) Transfer knowledge from natural language to electrocardiography: can we detect cardiovascular disease through language models? *arXiv* preprint arXiv:2301.09017. 10.48550/arXiv.2301.09017

[120] Greenberg JK , Frumkin M , Ziqi X , Zhang J , Javeed S , Zhang JK , Benedict B , Botterbush K , Yakdan S , Molina CA *et al*. (2024) Preoperative mobile health data improve predictions of recovery from lumbar spine surgery. Neurosurgery 95, 617–626. 38551340 10.1227/neu.0000000000002911

[121] Chung P , Fong CT , Walters AM , Aghaeepour N , Yetisgen M and O'Reilly‐Shah VN (2024) Large language model capabilities in perioperative risk prediction and prognostication. JAMA Surg 159, 928–937. 38837145 10.1001/jamasurg.2024.1621 PMC11154375

[122] Venkatesh KP , Brito G and Kamel Boulos MN (2024) Health digital twins in life science and health care innovation. Annu Rev Pharmacol Toxicol 64, 159–170. 37562495 10.1146/annurev-pharmtox-022123-022046

[123] Chauhan P , Bali A and Kaur S (2024) Breaking barriers for accessible health programs: the role of telemedicine in a global healthcare transformation. In Transformative Approaches to Patient Literacy and Healthcare Innovation ( Garcia MB and de Almeida RPP , eds), pp. 283–307. IGI Global, Hershey, PA.

[124] Chu Y , Li S , Tang J and Wu H (2023) The potential of the medical digital twin in diabetes management: a review. Front Med 10, 1178912. 10.3389/fmed.2023.1178912 PMC10397506 37547605

[125] Visan AI and Negut I (2024) Integrating artificial intelligence for drug discovery in the context of revolutionizing drug delivery. Life 14, 233. 38398742 10.3390/life14020233 PMC10890405

[126] Stahlberg EA , Abdel‐Rahman M , Aguilar B , Asadpoure A , Beckman RA , Borkon LL , Bryan JN , Cebulla CM , Chang YH , Chatterjee A *et al*. (2022) Exploring approaches for predictive cancer patient digital twins: opportunities for collaboration and innovation. Front Digit Health 4, 1007784. 36274654 10.3389/fdgth.2022.1007784 PMC9586248

[127] Li S , Moayedpour S , Li R , Bailey M , Riahi S , Kogler‐Anele L , Miladi M , Miner J , Zheng D , Wang J *et al*. (2023) Codonbert: large language models for mrna design and optimization. *bioRxiv* 2023‐09. 10.1101/2023.09.09.556981 [PREPRINT]

[128] Mao Y , Wang W , Yang J , Zhou X , Yongqu L , Gao J , Wang X , Wen L , Fu W and Tang F (2024) Drug repurposing screening and mechanism analysis based on human colorectal cancer organoids. Protein Cell 15, 285–304. 37345888 10.1093/procel/pwad038 PMC10984622

[129] Wang F , Wang H , Wang L , Haoyu L , Qiu S , Zang T , Zhang X and Yang H (2022) Mhcroberta: pan‐specific peptide–mhc class i binding prediction through transfer learning with label‐agnostic protein sequences. Brief Bioinform 23, bbab595. 35443027 10.1093/bib/bbab595

[130] Cheng J , Bendjama K , Rittner K and Malone B (2021) Bertmhc: improved mhc–peptide class ii interaction prediction with transformer and multiple instance learning. Bioinformatics 37, 4172–4179. 34096999 10.1093/bioinformatics/btab422 PMC9502151

[131] Olsen TH , Moal IH and Deane CM (2022) Ablang: an antibody language model for completing antibody sequences. Bioinform Adv 2, vbac046. 36699403 10.1093/bioadv/vbac046 PMC9710568

[132] Choi Y (2022) Artificial intelligence for antibody reading comprehension: Antiberta. Patterns (N Y) 3, 100535. 35845838 10.1016/j.patter.2022.100535 PMC9278504

